# Intrathrombus polymer coating deposition: a pilot study of 91 patients undergoing endovascular therapy for acute large vessel stroke. Part I: Histologic frequency

**DOI:** 10.1136/neurintsurg-2018-014684

**Published:** 2019-05-18

**Authors:** Rashi I Mehta, Ansaar T Rai, Jeffrey A Vos, Orestes E Solis, Rupal I Mehta

**Affiliations:** 1 Department of Radiology, West Virginia University, Morgantown, West Virginia, USA; 2 Department of Neurointerventional Radiology, West Virginia University, Morgantown, West Virginia, USA; 3 Department of Pathology, West Virginia University, Morgantown, West Virginia, USA; 4 Department of Pathology and Laboratory Medicine, University of Rochester, Rochester, New York, USA; 5 Department of Neuroscience, University of Rochester, Rochester, NY, USA

**Keywords:** complication, device, stroke, angiography, thrombectomy

## Abstract

**Background:**

Polymer coating embolism due to vascular medical device use is an increasingly recognized iatrogenic complication. This phenomenon has been linked with various adverse effects including neuroinflammation, acute ischemic stroke, cerebral hemorrhage, and death. Notably, procedure- and device-specific risks of this complication are poorly investigated. In this study, we evaluate the detectable frequency of intra-arterial polymer coating delamination among patients who underwent endovascular thrombectomy for treatment of acute ischemic stroke due to large vessel occlusion.

**Methods:**

Ninety-two cerebral thrombectomy specimens were retrospectively analyzed for the presence of polymer coating particulates. Histologic findings were correlated with demographic and procedural details and patient outcomes.

**Results:**

Evidence of polymer coating deposition was found in 30 of 92 extracted thrombi (33%). No correlation between intrathrombus polymer deposition and use of a specific thrombectomy device such as a stent retriever, aspiration catheter, or guide catheter was found. However, heterogeneous patterns of device use suggest a number of culprit devices. A trend toward longer procedure times and multiple thrombectomy passes was noted in positive cases. Intrathrombus polymer deposition was not associated with adverse clinical outcomes as measured by the 90-day modified Rankin Scale (mRS); however, small sample size and follow-up intervals limit interpretation. Ninety-day outcomes based on mRS may not fully capture the clinical effects of acute and/or delayed intracerebral polymer complications.

**Conclusion:**

In light of documented adverse neurologic effects, the frequency of intrathrombus polymer particulates indicates the need for consensus testing methods and large-scale long-term prospective clinical device trials, with inclusion of relevant endpoints to better assess biomaterial and device risks to patients.

## Introduction

Recent updates in the 2018 American Heart Association (AHA) guidelines for the management of patients with acute ischemic stroke add to growing trends in endovascular treatment of disease. The DAWN and DEFUSE 3 trials support endovascular thrombectomy for patients with emergent large vessel occlusion up to 24 hours after symptom onset,^1 2^ and greatly expand patient eligibility for this procedure. These trials mark a notable milestone in stroke management, and further expand the number of stroke patients who will be managed worldwide with endovascular devices.

Given the major trend, there is increased importance to assess device efficacy and safety. Iatrogenic polymer coating complications have gained attention among pathologists, regulators, and industry personnel over recent years.^3–9^ However, coating integrity and polymer delamination from device surfaces have not previously been evaluated among patients who undergo endovascular treatment of acute large vessel ischemic stroke. In this study we analyze the frequency of polymer coating deposition within cerebral thrombi retrieved via endovascular thrombectomy from patients with acute large vessel stroke, and correlate particulate characteristics with procedural and clinical data and patient outcome.

## Methods

### Study design

This study was performed in accordance with a protocol approved by West Virginia University’s institutional review board. Histopathologic evaluation of cerebral thrombectomy specimens has been routine at our institution since 2011. The pathology database was searched for cerebral thrombectomy specimens accessioned at our institution, a tertiary care hospital, and a recently designated comprehensive stroke center. All cases from 2011 through 2017 with available cerebral thrombus specimens retrievable from the archives were included in this retrospective analysis.

### Thrombectomy protocol

Endovascular thrombectomy was performed according to the routine institutional protocol on patients who presented with acute ischemic stroke secondary to large vessel occlusion, defined as occlusion of internal carotid artery terminus (ICA-T), middle cerebral artery main stem (M1), proximal bifurcation branches (M2), or basilar artery (BA). Device selection for cerebral thrombectomy procedures was at the discretion of the treating neurointerventional radiologist. Extracted thrombus specimens were submitted to pathology in 10% neutral buffered formalin. Following fixation, specimens were processed overnight, embedded in paraffin, sectioned at 5 µm thickness, mounted on glass slides, stained with hematoxylin and eosin (H&E), and coverslipped.

### Histologic evaluation of extracted thrombi

**Figure 1:**
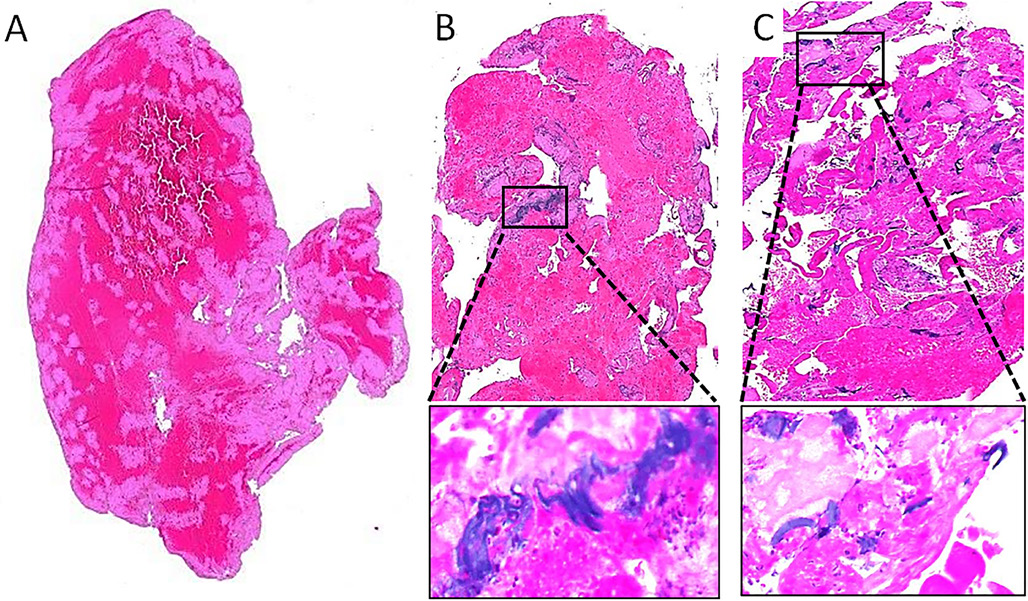
Histologic appearance of thrombi and coating deposits. (A) Thrombus without polymer particulates shows eosinophilic blood and fibrin products only. (B) Thrombus with embedded coating particulates, visualized as non-refractile basophilic foreign bodies measuring >100 µm in greatest dimension. (C) Thrombus with >100 scattered coating deposits. Boxed areas shown at high power (insets).

**Figure 2:**
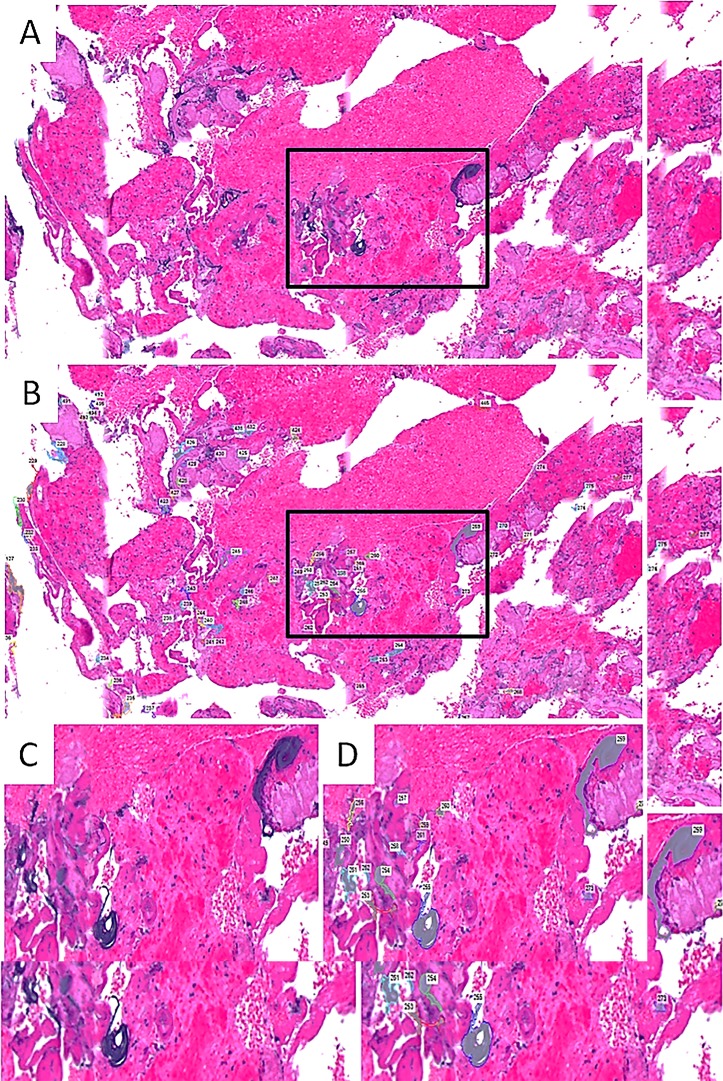
Quantitation of particulates. Coating particulates were identified on H&E-stained slides (A), and were manually traced by a neuropathologist using Nikon Microscope Solutions Imaging Software (NIS-Elements AR Version 4.30.01) to determine particulate counts and dimensions (B). Boxed areas show object count at higher power (C, D).

Retrospective histologic analysis was performed by two neuropathologists who were blinded to the procedural and clinical data. H&E-stained preparations of specimen materials were evaluated for the presence of foreign body polymer deposits, as previously characterized (figure 1).^5 10–12^ Analysis was conducted by light microscopic scanning of human tissue slides at 200× and 600× magnification. Particulate count, mean and maximal particulate cross-sectional areas (μm^2^), total particulate load (ie, total particulate cross-sectional area) (μm^2^), and total thrombus cross-sectional area (μm^2^) were determined in positive cases. Data acquisition was performed by digital tracing of particulates and thrombus specimens by two neuropathologists using Nikon Microscope Solutions Imaging Software (NIS-Elements AR Version 4.30.01) (figure 2).

### Review of procedural technique, patient demographics, and outcome

Patient charts were retrospectively analyzed by investigators who were blinded to the histopathologic data including the presence or absence of intrathrombus polymer particulates. Clinical and demographic data, including patient age, gender, National Institutes of Health Stroke Scale (NIHSS), patient outcome (death, 90-day modified Rankin Scale, TICI reperfusion grade), and length of stay, were recorded. Patient comorbidities including history of diabetes mellitus, hypertension, hyperlipidemia, atrial fibrillation, and smoking were determined. Additionally, procedural details including surgery duration, number of passes using a thrombectomy device, and use of specific devices (guidewire, guide catheter, microcatheter, aspirator, and stent retriever and/or embolectomy device) were recorded.

### Statistical analysis

Inter-rater agreement on the presence versus absence of polymer particulates was determined using Cohen’s kappa coefficient (κ). Using average particulate cross-sectional areas, mean radii and diameters of particulates were extrapolated and mean particle volumes were estimated, assuming average spherical morphology of particulates, using the formula V=4/3πr^3^. The presence and size characteristics of intrathrombus coating particulates were correlated with patient demographics, comorbidities, procedural technique, and patient outcome. All data analysis was performed using JMP statistical software, V.11 (SAS Institute, Cary, North Carolina, USA). The significance of simple bivariate associations was assessed using Fisher’s exact test for categorical variables, Student’s t-test for continuous variables, or logistic regression, as appropriate.

## Results

### Study population

Ninety-two cerebral thrombi retrieved from 91 patients were identified in our archives for inclusion in this study. Patient demographic and clinical data are summarized in table 1.

**Table 1:** Demographic and clinical information on 91 patients who underwent endovascular thrombectomy for acute ischemic stroke

Age, years	66±18
Female	49 (53%)
NIHSS	19 (IQR 11–23)
Thrombus location	
M1	55 (60%)
M2	3 (3%)
ICA-T	17 (18.5%)
BA	17 (18.5%)
Comorbidities	
DM	29 (32%)
HTN	62 (68%)
HL	44 (48%)
AFIB	39 (43%)
SMK	16 (18%)
Recanalization (≥TICI 2B)	78 (85%)
IV rt-PA administered	36 (39%)

AFIB, atrial fibrillation; BA, basilar artery; DM, diabetes mellitus; HL, hyperlipidemia; HTN, hypertension; ICA-T, internal carotid artery terminus; NIHSS, National Institutes of Health Stroke Scale; rt-PA, recombinant tissue plasminogen activator; SMK, smoking; TICI, Thrombolysis in Cerebral Infarction.

### Histologic analysis

**Figure 3:**
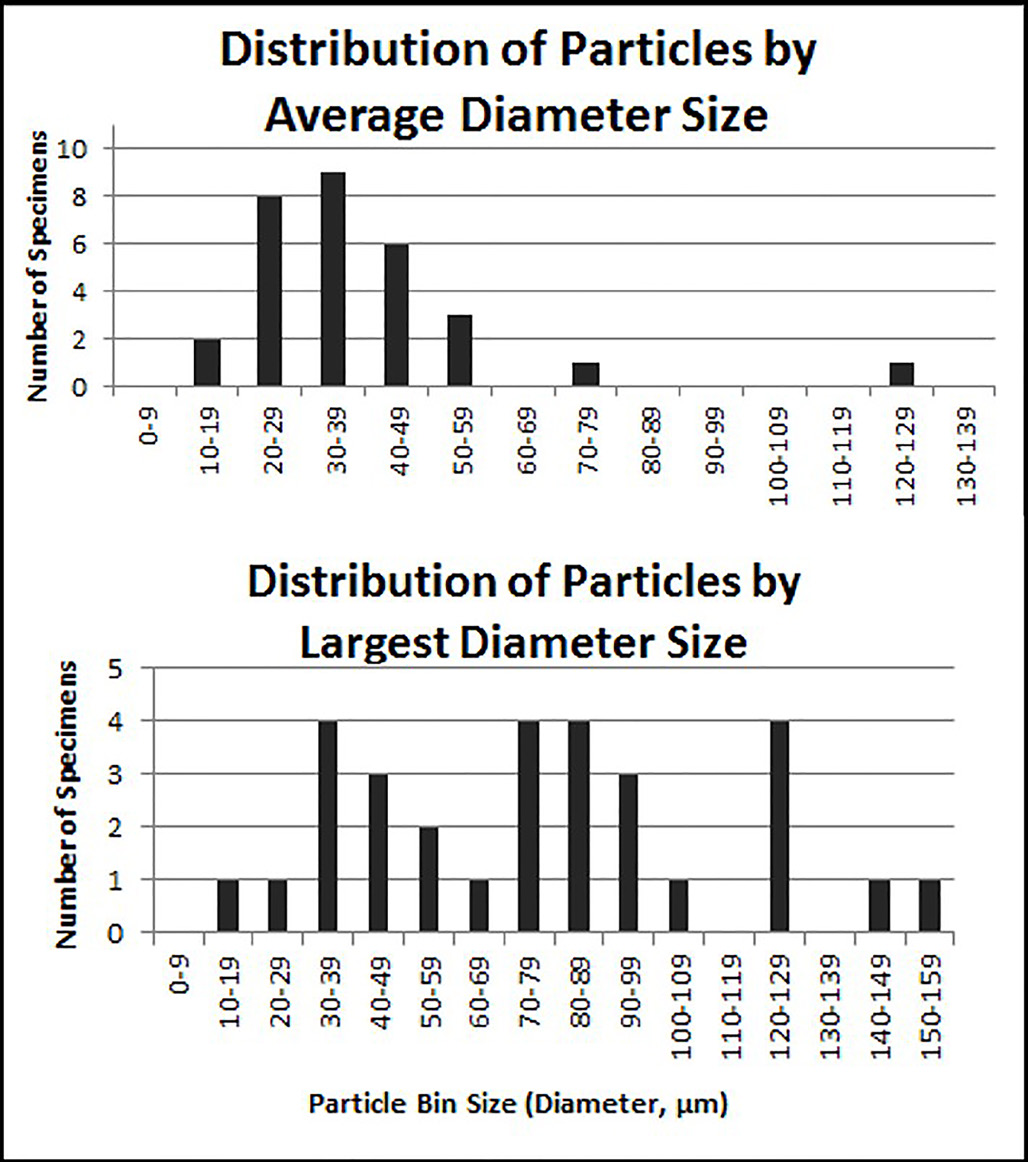
Distribution of average and largest particle size (diameter).

Delaminated polymer coating particulates were readily detected in 30 of the 92 cerebral thrombus specimens (33%). Deposits appeared as basophilic, granular, lamellar or amorphous, intrathrombus foreign bodies that were non-refractile and non-polarizable, as previously described (figure 1).^5 10–12^ Particulate counts, cross-sectional areas, overall load, mean and maximal particulate diameters, and estimated mean volume were variable, as summarized in table 2 and figure 3. No additional foreign material was identified in any case. All of the 30 positive specimens exhibited particulates measuring >100 µm^2^ in mean cross-sectional area; 21 (70%) exhibited particulates with maximum diameter >50 µm; 16 (53%) had particulate counts >10 in number and 1 (3%) had a total particulate count >100. Particulate counts ranged from 1 to 605. Specimens were independently reviewed for presence versus absence of polymer particulates by two experienced neuropathologists, with good inter-reader agreement (κ=0.95).

**Table 2:** Quantitative polymer characteristics in 30 specimens with evidence of intrathrombus polymer deposition

Quantitative polymer characteristics	Median (IQR)	Mean (SD)
Particulate count (per specimen, n)	11 (6–30)	37 (109)
Particulate diameter, mean (μm)	33 (28–45)	38 (20)
Particulate diameter, largest (μm)	75 (44–95)	74 (36)
Particulate area, mean per particle (μm^2^)	877 (590–1521)	1462 (2065)
Particulate area, total per specimen (μm^2^)	11084 (4601–30523)	29956 (68598)
Particulate to thrombus area ratio	0.07 (0.02–0.16)	0.19 (0.32)
Particulate volume, per particle (μm^3^)	19547	60737

### Correlation of intrathrombus particulates with patient outcome

There was no correlation between particulate counts, size, or burden with patient age or gender. A statistically significant correlation was not found between particulate deposition and patient outcome, using TICI reperfusion score, 90-day mRS, and mortality as outcome criteria. No significant correlation was identified between polymer delamination and comorbid conditions, including diabetes mellitus, hypertension, hyperlipidemia, atrial fibrillation, and smoking.

### Correlation of intrathrombus particulates with procedural technique

The presence of particulates trended with longer procedure times compared with cases in which no polymer was found (87±29 min vs 71±48 min, p=0.06). A trend was also seen with multiple thrombectomy passes. Particulates were present in 20 of 55 cases (36.3%) in which more than one thrombectomy pass was performed compared with 10 of 37 cases (27%) that required only a single pass (p=0.34). There was no statistically significant correlation between polymer delamination and specific stent retriever, aspirator device, catheter, or guidewire used. Additionally, use of a stent retriever was not found to generate greater polymer particulates compared with use of an aspirator catheter alone. Devices used in cases exhibiting evidence of intrathrombus coating particulates are listed in Table 3.

## Discussion

Microparticle embolism resulting from intravascular device use is an increasingly recognized complication with potential for significant unintended adverse neurologic effects. Particulates may be generated by suboptimal protocols for device manufacturing, packaging, storage, preparation, and/or operator use, and potential origins may include ambient materials (eg, dust, gauze fibers), native tissues (eg, dislodged myocardium or vascular wall elements), and/or intrinsic device materials such as device coatings. Due to complex in vivo responses, distinct particulate compositions warrant further investigation as each may present with unique organ effects and diagnostic challenges. Despite increasing use of endovascular devices, relatively few systematic analyses have analyzed the frequencies and characteristics of iatrogenic particulates, including risks of polymer coatings. In this investigation, one-third of cerebral thrombi (30 of 92 samples) mechanically retrieved from patients with acute large vessel ischemic stroke were found to contain delaminated device coating particulates. To our knowledge, this is the first investigation reporting frequency of device coating particulates among instrumented stroke patients.

The results of this study add to accruing evidence that intravascular polymer coating embolization occurs commonly during various endovascular procedures. A 2015 autopsy investigation of 136 patients demonstrated polymer coating deposits within the brain, lungs, and/or heart in 13% of adult hospital decedents who had undergone diverse cardiovascular interventions^8^; this postmortem study was not designed to evaluate device- or procedure-specific frequencies. In a separate analysis, Grundeken *et al* evaluated the frequency of polymer coating delamination associated with percutaneous cardiac revascularization among patients treated for acute myocardial ischemia.^7^ Retrospective analysis showed polymer particulates in 92 of 205 coronary thrombectomy specimens (45%), and were attributed to various cardiac guidewire coatings. In this same study, myocardium from patients treated with percutaneous coronary intervention were also evaluated on postmortem histology, revealing distal polymer emboli in 4 of 40 patients (10%). Rare prospective studies have assessed polymer coating delamination in live patients undergoing transcatheter cardiac valve and ablation surgeries, and show rates varying from 30% to 86% in these contexts.^13–16^ While evaluation of frequencies and clinical effects of polymer coating reactions in live patients presents complex challenges, available information on coating risks warrant continued investigation and establishment of new methodologies for device evaluation and testing.

Potential sequelae of embolized polymer coatings to the brain have previously been reported, and include death, ischemic and/or hemorrhagic stroke, chronic neuroinflammation, headache, constitutional symptoms, seizures, and cognitive dysfunction.^4–6 10–12 17–26^ While steroids and immunosuppressive therapy have been shown to be beneficial in some patients who develop inflammatory responses, long-term outcomes, subclinical inflammatory responses, and implications of comorbid small vessel diseases have not been systematically investigated. Furthermore, chronic neuroinflammatory reactions have persisted in some patients for several years, and have been associated with immunosuppressive-related adverse effects, white matter disease, cerebral volume loss, and secondary disability.^10 19 20 22 23^ Notably, these and other potential long-term tissue changes and organ effects would not be reflected through reporting on 90-day mRS of afflicted patients.

**Figure 4:**
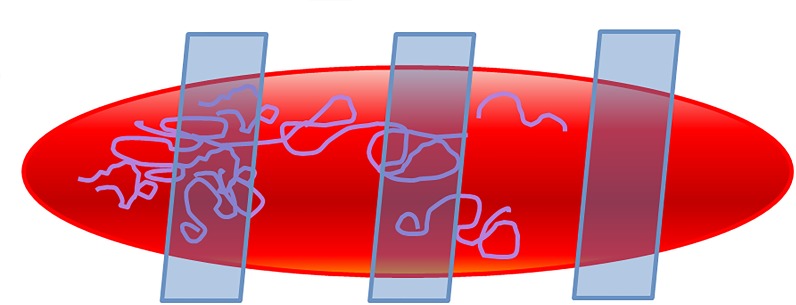
Limitations of testing method used. In the present study, a single random cross-section was assessed for the presence of coating particulates. As shown in the schematic, limited sampling and random sectioning may lead to false negatives and under-reporting of particulate burden.

Appropriate preclinical and clinical testing methods for device coating embolism remain undefined, in part related to complex morphologies and distributions of polymer particulates. This presents an impediment to understanding comparative risks of specific vascular medical device coatings. The current investigation along with other recent studies^13–16^ illustrate the feasibility of quantitatively assessing polymer particulates in live patients using common methodologies, although advantages and disadvantages of the testing methods used should be noted. Analysis of particulates is possible by performing routine histology on extracted thrombi without adding clinical risk to patients. Moreover, analysis of evacuated intravascular tissues (eg, thrombectomy and filtration-based specimens) likely yield greater sensitivity than downstream tissue sampling and provide greater insight on the causative procedure. However, histologic testing methods are associated with false negatives, as random tissue sampling and evaluation of single 5 µm thick sections allow only for screening of limited portions of patient specimens. Thrombus specimens assessed in this investigation were not analyzed volumetrically, nor in their entirety (figure 4). Furthermore, coating fragments liberated into the bloodstream and deposited in downstream sites could not be evaluated in the current study. Therefore, distal tissue responses, including ischemic and inflammatory sequelae, cannot be evaluated with this technique and would require invasive biopsy or postmortem analysis. Identification of specific polymer chemistry is not presented in the current report, and requires additional chemical particle analysis (eg, with Fourier transform infrared or Raman spectroscopy). As particulate deposits were not prospectively diagnosed in any of the 30 positive thrombus samples tested here, detection of polymer deposition in human tissues requires knowledge on this complication, an index of suspicion, targeted tissue sampling, and high-power microscopic analysis^4^; these factors each impact on test sensitivity. Despite this, good inter-reader agreement was observed in this retrospective analysis.

Consensus methods for detection, quantification, and reporting of polymer particulates in tissue specimens are needed and would allow for more meaningful analyses of embolic coating complications. As summarized here, different approaches may be taken to quantify and report particle loads (ie, one-dimensional, two-dimensional, three-dimensional particle analysis), with measurements of maximum one-dimensional length not fully capturing particulate size and burden, and actual particulate volumes not obtainable through evaluation of single histologic sections. While coating particulates vary widely in shape and size, extrapolated particulate volume and mean diameter analysis based on average spherical morphologies may be a useful technique to estimate and report polymer burdens in actual patient specimens, and may be more informative for comparative in vitro and regulatory device testing.

Device-specific particulate generation would best be measured through in vitro and ex vivo device surface analyses following use in simulated or real-world environments. Despite the limited ability for in vivo analyses to yield device-specific information, clinical studies remain critical to evaluate actual effects and particulate burdens associated with various device use in patients. In this investigation, a correlation between polymer delamination and use of a stent retriever or specific thrombectomy device, catheter, or guidewire was not identified. However, due to sample size and heterogeneous patterns of device use, this study was not designed or powered to make any definite conclusions regarding specific device-associated risks. It is now recognized that a number of devices contribute to intravascular polymer particulate generation, and the results of this study further suggest a need for novel methods of inspection of coated devices intended for intravascular clinical use. Although no correlation between polymer delamination and adverse patient outcome was identified in this investigation, 90-day mRS alone is not an appropriate measure for assessment of clinical significance. For future investigation, more standardized methods of clinical testing and formalized reporting of particulate size, loads, and intravascular effects has potential to impact on future guidance for biomaterial selection and standards.^4 27^

## Conclusions

Coating particulate microembolism occurs commonly during endovascular procedures; however, quantitation of frequencies and risks of associated complications are underinvestigated. Furthermore, existing evidence shows that current preclinical simulated use testing is not fully predictive of device coating performances in real-world settings.^4^ To better understand the safety and integrity of device surface materials and procedural techniques, appropriate endpoints must be established and incorporated into preclinical testing and clinical trials designed to evaluate acceptable standards of device manufacturing and biomaterial use. The importance of expanded preclinical in vitro safety testing has been acknowledged by the FDA,^3^ but further clinical investigation is needed and will likely drive preclinical testing requirements and industry standards.^4^ Large-scale, multi-institutional clinical device and tissue registries with long-term follow-up would facilitate characterization and comparative analyses of specific biomaterial and device effects in live patients, and will help identify source coatings. While there has been over time a generalized increase in awareness of the importance of studying particulates, formal preclinical and clinical guidelines and consensus recommendations for testing, interpretation, and reporting are lacking and remain an important priority area for determination of comparative procedure and device safety.

**Table 3:** Devices used in cases with evidence of intrathrombus coating particulates

Case	Occlusion site	Passes	Guide	Catheters	Wires	SR	Other
1	BA	4	Chaperon	P3-Max	F16		Hyperglide
2	BA	4	Benchmark	P3-Max, MM	Avigo^3^	SOL4×20	Coronary stent
3	BA	3	NMAx	P5-Max, MM	F16	SOL6×30	
4	BA	2	Infinity	Cat-6, PP	Synchro^2^	SOL4×20	
5	BA	2	NMAx	P64, PP	Avigo	SOL4×20	
6	ICA-T	4	Infinity	Cat-6, Sophia, XT-27	Synchro	TP4×30, 6×30	
7	ICA-T	4	NMAx	P3-Max, MM, PP	Avigo	SOL4×20	Protégé stent
8	ICA-T	4	Flowgate	Cat-6, MM	Synchro	SOL6×30	
9	ICA-T	3	Infinity	P5-Max, XT-27	Synchro	TP6×25	Protégé stent
10	ICA-T	2	NMAx	P3-Max, P68	Synchro		Protégé stent
11	ICA-T	1	Flowgate	MM	F16	SOL6×30	
12	ICA-T	1	Flowgate	MM	F16	SOL6×30	
13	ICA-T	1	SS	P3-Max, P5-Max	F16		
14	ICA-T	1	Flowgate	MM	F16	SOL6×30	
15	M1	4	Flowgate	MM, XT-27	F16	TP4×30, SOL6×30	
16	M1	3	Flowgate	MM	F16	SOL6×30	
17	M1	3	Flowgate	MM, PP	Avigo, Synchro	SOL6×30, 4×20	
18	M1	3	Flowgate	XT-27	Synchro	TP6×25, SOL6×30	
19	M1	2	NMAx, SS	P3-Max, P64	F16		
20	M1	2	Flowgate	MM/PP	F16, Avigo	SOL4×20	
21	M1	2	Infinity	P3-Max, P68	F16		
22	M1	2	NMAx	P5-Max, P3-Max, XT-27	Synchro	TP6×20	
23	M1	2	Infinity	P3-Max, P68	F16		
24	M1	1	Flowgate	XT 27	F16	TP6×25	
25	M1	1	Flowgate	MM	F16	SOL6×30	
26	M1	1	Flowgate	MM	F16	SOL6×30	
27	M1	1	SS	MM	Avigo	SOL6×30	
28	M1	1	Flowgate	PP	Avigo	SOL4×20	
29	M1	1	Flowgate	MM	F16	SOL6×30	
30	M2	2	Flowgate	MM	F16, Avigo	SOL4×20	

BA, basilar artery; ICA-T, internal carotid artery terminus; SR, stent retriever.

Avigo PP, Prowler Plus^6^; Cat-6, Catalyst-6^2^; F16, Fathom 16^4^; Infinity, AXS Infinity LS^2^; MM, Marksman^3^; NMax, NMAx^1^; P3-Max, Penumbra 3 Max^1^; P5-Max, Penumbra 5 Max^1^; P64, Penumbra ACE 64^1^; P68, Penumbra ACE 68^1^; SOL, Solitaire^3^; SS, Shuttle Sheath; TP, Trevo ProVue^2^; XT-27, Excelsior XT-27^2^.

1 Penumbra, Alameda California, USA; ^2^Stryker Neurovascular, Fremont California, USA; ^3^Medtronic Neurovascular, Irvine California, USA; ^4^Boston Scientific, Marlborough, Massachusetts, USA; ^5^Microvention, Aliso Viejo, California, USA; ^6^Cerenovus, New Brunswick New Jersey, USA.
